# A role for the Epstein–Barr virus in multiple sclerosis aetiology?

**DOI:** 10.1007/s00415-022-11177-w

**Published:** 2022-05-31

**Authors:** J. Hrastelj, N. P. Robertson

**Affiliations:** 1 Department of Neurology, University Hospital of Wales, Heath Park, Cardiff, CF14 4XN UK; 2 Department of Neurology, Division of Psychological Medicine and Clinical Neuroscience, Cardiff University, University Hospital of Wales, Heath Park, Cardiff, CF14 4XN UK

## Introduction

The association between Epstein–Barr virus (EBV) and multiple sclerosis (MS) has been known for many years. EBV seropositivity in MS has been reported as 99.5–100% compared with ~ 94% in healthy adults, and MS is over twice as common in people who have had symptomatic infectious mononucleosis. Furthermore, patients with MS also have elevated antibodies to EBV nuclear antigens (EBNAs), and EBV has been isolated from MS demyelinating plaques. However, a causal relationship has yet to be fully established.

The first paper discussed this month reports a striking temporal relationship between EBV seroconversion and MS onset in a large US military cohort. In the second paper, evidence is presented that suggests clonally expanded B cells in the CSF produce oligoclonal bands, as well as antibodies that cross-react with proteins expressed by EBV and oligodendrocytes. The final paper discussed this month reports that an EBV transcription factor occupies a large number of the MS genetic risk variants and this may at least partly explain how EBV infection and genetic risk intersect to cause MS.

## Longitudinal analysis reveals high prevalence of Epstein–Barr virus associated with multiple sclerosis

There is now a large body of evidence supporting a link between EBV infection and MS, but the direction of causality has been difficult to confirm. Bjornevik et al. report the results of a 20-year cohort study of over 10 million active US military personnel between 1993 and 2013. Personnel were screened for HIV on a biennial basis during their service and residual serum was used to screen for EBV seropositivity. They identified 955 incident cases of MS, 801 of which had adequate serum samples available. For each case, three serum samples were identified: the first available, the sample immediately before MS onset and one in between. Two matched controls were identified for each MS case.

Individuals with EBV at baseline were more than 26 times more likely to develop MS during the study period. Individuals that seroconverted during the study period were more than 32 times more likely to develop MS. All but one case seroconverted before the clinical onset of MS, with a median delay of 7.5 years. To assess whether a confounder could independently lead to EBV infection and MS, seropositivity to CMV was tested, because CMV infection displays very similar epidemiology to EBV infection. There was no increased risk of MS in those who were CMV positive.

As it is accepted that a preclinical phase of inflammation occurs in MS, the authors explored the temporal relationship between EBV seropositivity and a marker of neuronal injury, neurofilament light chain (NfL). There was no difference in NfL levels between cases and controls before or at the time of EBV seroconversion, but levels were significantly elevated in samples taken after EBV seroconversion in individuals that later developed MS. Therefore, EBV seroconversion preceded the elevation in NfL levels in individuals that went on to develop MS.

Finally, the authors screened the samples for seropositivity to all known human virus (> 200 species) to explore the role of other infections in MS aetiology. There were no differences in any other viral infections between cases and controls.

***Comment:*** The authors carefully utilised a very large cohort of US military to clearly show that EBV seroconversion is strongly associated with MS and precedes its onset. This study provides compelling evidence for a causal relationship for EBV infection in MS and will hopefully provide impetus to efforts to elucidate the causative mechanisms, as well as to vaccine development.

*Bjornevik K et al. (2022) Science 375(6578):296–301*.

## Clonally expanded B cells in multiple sclerosis bind EBV EBNA1 and GlialCAM

Molecular mimicry between EBV proteins and proteins in the central nervous system has been hypothesised to be the mechanism underpinning autoimmunity in MS. Antibodies to EBNA1 have been detected in nearly all patients with MS before the onset of clinical symptoms.

Lanz et al. found that B cells in the CSF of patients with MS were dominated by a few clones of plasmablasts that have undergone somatic hypermutation in response to antigen, and that these are the source of CSF oligoclonal bands. Consistent with previous studies, they also showed that CSF B cell-encoded antibodies bind a specific region of EBNA1. This region of EBNA1 was then found to mimic a region of GlialCAM, a cell adhesion molecule expressed by astrocytes and oligodendrocytes. Multiple EBNA1-binding antibodies produced by CSF B cells in patients with MS were shown to cross-react to GlialCAM with high affinity. Cross-reacting antibodies were also detected in plasma of patients with MS.

The group then went on to show that immunisation with EBNA1 exacerbated the mouse model of MS, experimental autoimmune encephalomyelitis (EAE). Immunisation with EBNA1 before induction of EAE induced cross-reactive antibodies against EBNA1 and GlialCAM in the mice, as well as a substantially stronger CD4^+^ T cell and cytokine response. The EBNA1-immunised mice developed more severe demyelination and weakness.

***Comment:*** This study demonstrated that CSF oligoclonal bands are produced by clonally expanded B cells. These B cells produce antibodies that cross-react with EBNA1 and GlialCAM, which could be the mechanism that underpins demyelination. Although the numbers included were small, the results build on previous studies to provide robust evidence that antibody cross-reactivity with an EBV protein and a central nervous system antigen is important in MS pathogenesis.

*Lanz TV et al. (2022) Nature 603:321–7*.

## Transcription factors operate across disease loci, with EBNA2 implicated in autoimmunity

EBV infection appears necessary, but not sufficient to cause MS  and approximately 50% of the risk for MS is genetic. The mechanisms by which genetic risk variants for MS lead to disease are yet to be fully understood. One of the main reasons for this is that the risk variants are largely in non-coding genomic sequences, so do not alter protein amino acid sequence. Instead, it is likely that such variants alter gene expression in disease-relevant cells.

Harley et al. use a new computational method to identify which transcription factors bind to genetic risk variants for a range of disorders. They identified that the EBV transcription factor, EBNA2, occupies 44 of the 109 tested genetic risk variants for MS in EBV-infected B cells. Strong associations were also observed for eight other disorders, including systemic lupus erythematosis, rheumatoid arthritis, inflammatory bowel disease and type 1 diabetes. MS genetic risk loci were also frequently occupied by transcription factors associated with NF-ĸB signalling, which has been implicated in MS pathogenesis by multiple lines of evidence. Almost 30% of the EBNA2-bound genetic risk loci were shown to alter gene expression. Furthermore, EBNA2 and other transcription factors were found to preferentially bind one allele of a risk variant over another, suggesting that genetic variation at the MS risk loci may influence EBNA2-binding.

When the group analysed other cell types, such as T cells, they showed that the number of MS genetic risk loci occupied by transcription factors was highest in EBV-infected B cells. This suggests that genetic risk for MS carried by the tested variants is most likely to be mediated through altering the function of EBV-infected B cells.

***Comment:*** How EBV infection and genetic risk factors combine to cause MS is incompletely understood. This study demonstrated that EBNA2 occupies a large fraction of the MS genetic risk variants and this alters gene expression in EBV-infected B cells and drives NF-ĸB signalling. Genetic variation at the risk loci was shown to affect EBNA2-binding, suggesting that a proportion of the genetic risk for MS is likely mediated through altering how EBV disrupts gene expression when it infects B cells.

*Harley JB et al. (2018) Nature Genetics 50:699–707*.

